# The necessary pathway for metabolic and crystallographic analysis of kidney stones: struvite may not differ from its counterparts

**DOI:** 10.1590/2175-8239-JBN-2021-E004

**Published:** 2021-05-24

**Authors:** Igor Pietrobom, Ita Pfeferman Heilberg

**Affiliations:** 1 Universidade Federal de São Paulo, Escola Paulista de Medicina, São Paulo, SP, Brasil.

Struvite stones represent the most common cause of the rapidly growing staghorn calculi whose predisposing factors include female sex, stasis from urinary tract malformations or obstruction, neurogenic bladder, among others^1^. Their relevant morbidity is ascribed to the frequent association with recurrent urinary tract infections (UTI), pyelonephritis, perinephric abscess or even sepsis, not to mention the potential loss of renal function^2^. Moreover, lower social economic status and poor health care quality are closely related to reduced access to procedures for stone removal, lower rates of preventive management, and delayed or no treatment of recurrent UTI, altogether predisposing to the formation of infection stones. Accordingly, Cunha et al.^2^ have recently shown that struvite calculi are most commonly observed in areas of low human development indexes (HDI). As a consequence, patients may undergo radical nephrectomy more frequently compared to patients with other type of stones. Finally, there seems to be a greater chance of progressive renal dysfunction or chronic kidney disease (CKD) as a result of nephrectomy among struvite stone-forming patients, obstructive uropathy, or recurrent UTI^3^.

We read with great interest the work of Danilovic et al^4^, who conducted a retrospective evaluation of the charts of patients submitted to unilateral total nephrectomy due to pure MAP (magnesium ammonium phosphate or struvite stones) or pure calcium oxalate (CaOx) stones, searching for urinary metabolic parameters predictive of new clinical events. Although this topic has been raised in recent years^1^, the lack of data in our population highlights the epidemiologic relevance of their study^4^.

The authors found a similar frequency of metabolic abnormalities in 24-hour urine tests between groups, but only important for hypocitraturia. There was no significant difference in new events between groups, and treatment of metabolic abnormalities among patients with MAP stones rendered them prone to the same risk for a new event as those without any metabolic disturbance.

Although metabolic abnormalities in pure struvite stone formers indeed appear to be more common than previously reported, the profile of metabolic alterations differ among reports. Iqbal et al^1^, for instance, reported much higher rates of hypercalciuria (43%) and lower rates of hypocitraturia (14%) among pure struvite stone formers than the ones presented by Danilovic et al^4^. A possible reason could have been a sample with approximately a third of patients in stage 3 CKD in both groups (CaOx and MAP) by the latter. Moreover, there are limitations in urinary metabolic evaluation of patients with glomerular filtration rates (GFR) lower than 60 mL/min/1.73m^2^, especially concerning lower rates of urinary calcium, due to secondary hyperparathyroidism, as already demonstrated in primary struvite urolithiasis populations^5^. The authors also did not find differences in citrate excretion between MAP and CaOx groups although urinary citrate has been shown to correlate with eGFR^6^. Therefore, the metabolic evaluation in this group of patients must be interpreted with caution. Although potassium citrate supplementation may help prevent struvite crystallization, overt alkalinization must be averted. Due to the retrospective characteristic of the study, urinary pH values were unfortunately not available before or after nephrectomy. Another concern highlighted by the authors was the chemical analysis of the calculi, which might have hampered their analysis, providing inaccurate reporting of mineral composition. Kidney stones should preferably be analyzed by a physical method, namely a crystallographic analysis, typically performed by either infrared spectroscopy (IRS) or X-ray diffraction (XRD) to better identify mineral types and even distinguish between brushite from other forms of calcium phosphate or pure MAP^7^.

Nevertheless, the study by Danilovic et al^4^ sheds light on the management of kidney stones in the ambulatory setting. One should consider frequent evaluation of the medical history and physical examination, stone analysis by XRD or IRS in different time frames, and periodic metabolic evaluation based on 24-h urine samples^5^ especially in cases that need surgical procedures and in patients with altered clinical manifestations and social or geographic changes in the time course of follow up, as these factors may affect management overtime.
